# Interscalene brachial plexus block for surgical repair of clavicle fracture: a matched case-controlled study

**DOI:** 10.1186/s12871-020-01005-x

**Published:** 2020-04-20

**Authors:** Magnus Olofsson, Patrick Taffé, Kyle Robert Kirkham, Frédéric Vauclair, Bénédict Morin, Eric Albrecht

**Affiliations:** 1 Department of Anaesthesia, Lausanne University Hospital, Rue du Bugnon 46, BH 05.311, 1011 Lausanne, Switzerland; 2 Institute of Social and Preventive Medicine (IUMSP), Lausanne University Hospital, Lausanne, Switzerland; 3 Department of Anaesthesia, Toronto Western Hospital, University of Toronto, Toronto, Canada; 4 Department of Orthopaedic, Lausanne University Hospital, Lausanne, Switzerland

**Keywords:** Clavicle, Locoregional anaesthesia, Pain, Surgery, Postoperative, Ultrasound, Brachial plexus

## Abstract

**Background:**

Innervation of the clavicle is complex and debated, with scarce data on the analgesic and clinical impact of regional anaesthesia after surgical repair of clavicle fracture.

**Methods:**

In order to assess the analgesic efficiency of an interscalene brachial plexus block (ISB) for surgical repair of clavicle fracture, 50 consecutive patients scheduled for surgical fixation of middle/lateral clavicle fracture under general anaesthesia with ISB were prospectively enrolled. This cohort was compared to a historical control of 76 retrospective patients without regional block. The primary outcome was total intravenous morphine equivalent consumption at 2 postoperative hours. To assess the ISB impact, both an overall cohort analysis and a case-matched analysis with each ISB-treated patient matched to a Non-ISB-treated patient was performed. Matching employed a 1-to-1, nearest-neighbour approach using the Mahalanobis metric.

**Results:**

In the overall cohort, patients with ISB had significantly lower i.v. morphine equivalent consumption at 2 postoperative hours (0.7 mg (95% CI 0.1 to 1.2) versus controls 8.8 mg (95% CI 7.1 to 10.4); *P* <  0.0001). These results persisted after case-matching the cohorts (mean difference for the primary outcome: 8.3 mg (95% CI 6.5 to 10.0); *P* <  0.001).

**Conclusions:**

ISB provides effective analgesia after surgical fixation of middle and lateral clavicle fracture. These results should help physicians in establishing an analgesic strategy for this type of surgery. Further research is needed to identify the optimal regional technique for medial third clavicle fractures and the clinically relevant contributions of the cervical and brachial plexus.

**Trial registration:**

Clinicaltrials.gov – NCT02565342, October 1st 2015.

## Background

Surgical fixation of clavicular fractures may result in moderate to severe postoperative pain that does not always respond well to opioid therapy. If effective, a regional technique may therefore represent an analgesic improvement with the potential to reduce postoperative opioid consumption [1–3]. However, innervation of the clavicle remains a source of much debate. A recent report illustrated the state of current anatomic knowledge on this topic suggesting that contributions might come from: the cervical plexus through the supraclavicular nerve or from the brachial plexus with contributions from the subclavian nerve, the long thoracic nerve or the suprascapular nerve [4]. This anatomic uncertainty means anaesthetists struggle to determine the optimal analgesic strategy [4–6]. Furthermore, to date there has been no study that evaluates the analgesic efficacy of an interscalene brachial plexus block in patients with clavicular fractures. With the goal to resolve this clinical dilemma, we undertook a matched case-control cohort study assessing the analgesic impact of ultrasound-guided interscalene brachial plexus block (US-ISB) for patients scheduled for open reduction and internal fixation (ORIF) of middle or lateral clavicle fracture.

## Methods

We followed the recommended process described in the Strengthening the Reporting of Observational Studies in Epidemiology (STROBE) statement [7].

### Recruitment

After approval by the Lausanne University Hospital Ethics Committee (Commission d’Ethique Romande, protocol number CHUV 317/15, Chairperson Prof. André Pannatier) on 26th October 2015, this study was prospectively registered on clinicaltrials.gov (NCT02565342). All patients aged 18 to 70 years, American Society of Anesthesiologists score (ASA) I-II, scheduled for middle or lateral clavicle fracture ORIF at the Lausanne University Hospital were eligible to participate in this study. Exclusion criteria included existing neurological deficit in the upper limb, history of neck surgery or radiotherapy, moderate to severe pulmonary disease, contraindications to peripheral nerve block (e.g., allergy to local anaesthetics, coagulopathy, infection in the area), pre-existing opioid treatment, any distracting pain (i.e. polytraumatized patients), pregnancy and cognitive or psychiatric condition that might affect patient assessment. All surgeries were performed electively. Written informed consent was obtained prior to the day of surgery.

### Ultrasound-guided interscalene brachial plexus block

All US-ISB were performed prior to surgery in a dedicated block procedure room, following an extrafascial approach without nerve stimulation [8–10]. These blocks were administered or directly supervised by one of the authors (EA) who had no further involvement in the study protocol. Patients were positioned supine with the head turned 45 degrees to the non-operative side. Electrocardiogram, pulse oximetry, and blood pressure monitors were routinely applied, and supplemental oxygen was provided. Peripheral intravenous (i.v.) access was established and midazolam 1 to 4 mg i.v. was administered for anxiolysis and sedation as needed. The needle insertion site was sterilized with a solution of chlorhexidine 2% in isopropyl alcohol 70%. Under sterile conditions, a high-frequency linear array transducer (18–6 MHz, HF Linear Array 8870, BK Ultrasound, Peabody, Massachusetts) was placed over the interscalene region to visualize the carotid artery and brachial plexus in the short axis view. The C5, C6, and C7 roots were identified as described by Martinoli and colleagues [11]. After skin infiltration with 1 to 3 mL of lidocaine 1%, a 22-gauge 50-mm insulated block needle (SonoPlex Stim cannula, Pajunk®, Geisingen, Germany) was inserted in-plane with the US beam on the lateral side of the transducer. The needle was then advanced under direct US guidance through the middle scalene muscle and toward the lateral border of the brachial plexus sheath. The brachial plexus sheath was identified as the linear hyperechoic layer surrounding the roots of the brachial plexus. The final needle tip was positioned extrafascially, about 3 to 5 mm laterally to the brachial plexus sheath, at a depth equidistant between C5 and C6 roots. All patients received 20 mL of bupivacaine 0.5% with epinephrine 1:200,000 through the block needle without repositioning, except in cases of reported paraesthesia.

### Intraoperative and postoperative procedure

After application of routine monitors in the operating theatre, patients received a standard general anaesthetic. Anaesthesia was induced using Sufentanil 0.1 to 0.2 μg kg^− 1^ i.v. and Propofol 2 to 4 mg kg^− 1^ i.v. with endotracheal intubation facilitated by rocuronium 0.6 mg kg^− 1^ i.v. Maintenance of anaesthesia was via inhaled sevoflurane 1.6 to 2.4% in a 40:60 mixture of oxygen and air. Positive pressure ventilation was initiated with tidal volume and rate adjusted to maintain an end-tidal PCO_2_ of 35 to 40 mmHg. Sufentanil 2.5–5.0 μg i.v. was administered as needed to treat increases in blood pressure or heart rate of more than 15% above pre-induction baseline values. Muscle relaxation was antagonized with neostigmine 50 μg kg^− 1^ and glycopyrrolate 5 to 10 μg kg^− 1^ at the end of surgery. In the Post-Anesthesia Care Unit (PACU), pain (numeric rating scale [NRS] ≥ 4 or patient request for analgesia) was treated with i.v. morphine 1–2 mg every 10 min as needed for 2 h following our institutional procedure. Once oral intake was initiated, patients received oral acetaminophen 1000 mg every 6 h and oxycodone 5 mg every 4 h as needed. Antiemetic medications on the ward included ondansetron 4 mg i.v. and metoclopramide 10 mg i.v. as needed.

### Block assessment and definition of successful block

Assessment of sensory and motor blocks was performed by a research assistant every 5 min after local anaesthetic injection, for a total duration of 30 min. Sensory block was tested in the C5 and C6 dermatomes using a blunt tip needle pin-prick test (0, no perception; 1, decreased sensation; 2, normal sensation). Motor block was tested using arm abduction (C5), and forearm flexion (C6) (inability to overcome gravity, 0; reduced force compared to contralateral arm, 1; no loss of force, 2). A successful block was defined as complete sensory (score, 0) and motor (score, 0) block in the distribution of the C5 and C6 nerve roots within 30 min of performing the US-ISB block.

### Outcomes

The primary outcome was total i.v. morphine consumption at 2 postoperative hours upon departure from the PACU. Secondary outcomes were intraoperative Sufentanil administration; i.v. morphine equivalent consumption at 24 postoperative hours; pain scores at rest (NRS 0–10) at 2 and 24 postoperative hours; and rate of postoperative nausea and vomiting (PONV) within 24 postoperative hours. Opioids were converted into equianalgesic doses of i.v. morphine for analysis (i.v. morphine 10 mg = oral oxycodone 20 mg) [3, 12].

### Control cohort selection

All patients aged 18 to 70 years old, ASA score I-II, who had undergone middle or lateral clavicle fracture ORIF under general anaesthesia only, between September 2012 and August 2015 at the same institution as this study was conducted, were included in the historical control cohort. Exclusion criteria included pre-existing opioid tolerance, any distracting pain (i.e. polytraumatized patients), pregnancy and cognitive or psychiatric condition that might affect patient pain assessment. All surgeries were performed electively. The data was collected using the surgical calendar software in use at our institution.

### Statistical analysis and matching procedure

Categorical variables are presented as frequencies and continuous variables are summarized as mean values with 95% confidence intervals (95% CI). In the preliminary analysis, ISB-treated and Non-ISB-treated patients were compared using the Student’s t test or Mann–Whitney U test for continuous variables, and the Fisher’s exact test or Pearson Chi-square test for categorical variables, as appropriate. To assess the impact of the US-ISB procedure on the outcomes, we matched each ISB-treated patient with a Non-ISB-treated patient and computed the difference in means. The matching procedure was 1-to-1 nearest-neighbour matching using the Mahalanobis metric [13]. Therefore, for each exposed (ISB) individual, one unexposed (Non-ISB) individual, having the smallest possible Mahalanobis distance between the two vectors of covariates, (patients’ and intervention characteristics), was selected, and reversely for each non-exposed individual. Patients characteristics considered for the matching procedure were the gender, the age, the body mass index, the ASA score, the fracture location, the total dose of Propofol at induction and the duration of surgery. The standardized mean differences were computed for each variable before and after matching to assess the performance of the matching procedure (i.e. balance checking). We also used a logistic regression approach to assess whether some variables (gender, age, body mass index, ASA score, fracture location) were associated with the allocation of US-ISB. Significance was considered at *P* < 0.05 based on a two-tailed probability. Statistical analyses were performed using the Stata 15 statistical package (Stata Corporation, College Station, Texas, U.S.A.).

## Results

Fifty patients with an US-ISB were prospectively included and compared with 76 patients who did not receive an interscalene brachial plexus block. All US-ISBs attempted were successful. Table 1 presents patients’ characteristics.

**Table 1:** Patient characteristics and clinical data presented as means (95% confidence interval) or percentages as appropriate

	Control group (*n* = 76)	US-ISB group (*n* = 50)	*p* value
Gender (male / female)	82% / 18%	84% / 16%	0.73
Age (years)	35 (32–38)	36 (32–41)	0.66
Height (cm)	177 (175–179)	177 (174–180)	0.94
Weight (kg)	74 (71–76)	75 (71–78)	0.67
Body Mass Index (kg.m^− 2^)	23.4 (22.7–24.1)	23.6 (22.8–24.4)	0.67
ASA (I / II)	53% / 47%	50% / 50%	0.77
Fracture location (middle / distal)	78% / 22%	72% / 28%	0.47
Total dose of Propofol at induction (mg)	249 (231–267)	265 (242–287)	0.29
Duration of surgery (minutes)	96 (89–104)	101 (94–108)	0.35

*ASA* American Society of Anaesthesiologists

### Primary outcome

**Fig. 1:**
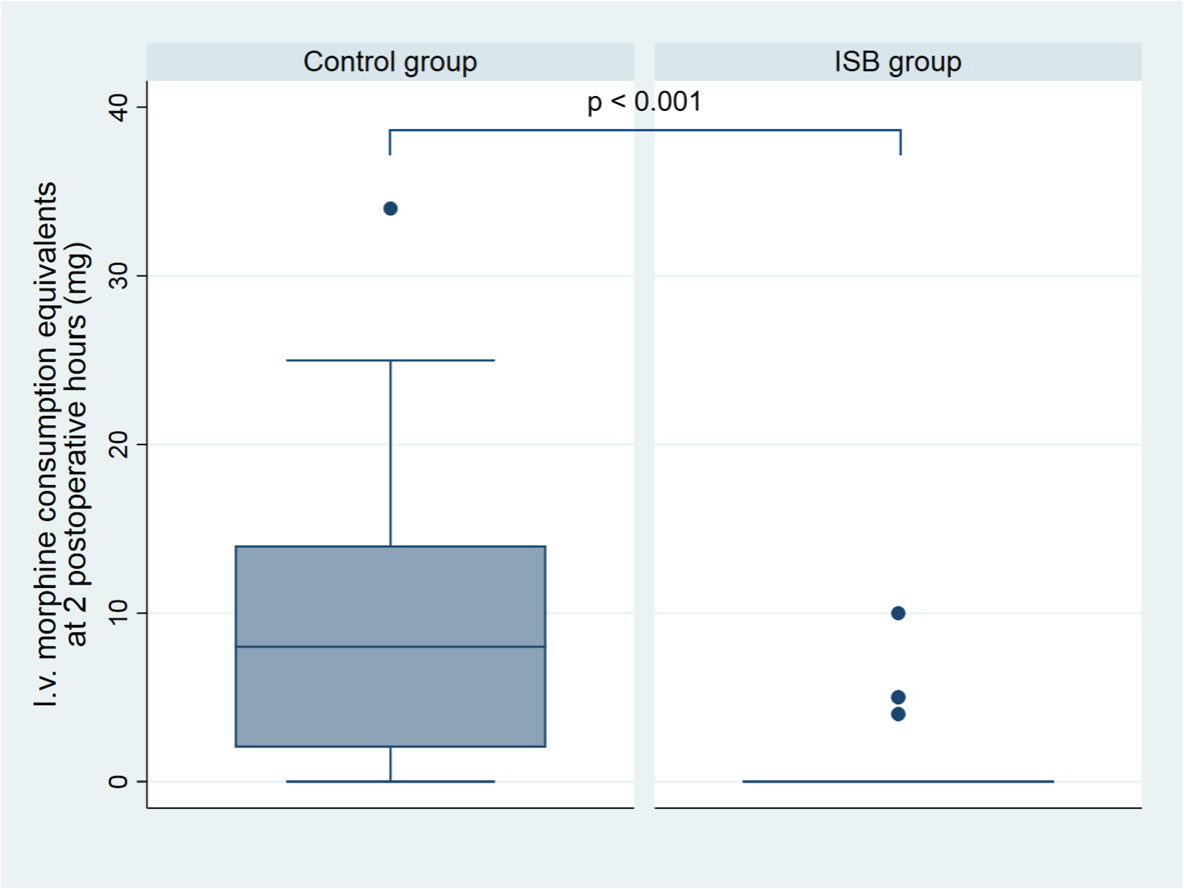
I.v. morphine consumption equivalents at 2 postoperative hours (mg). Data are expressed as the median with 25th and 75th percentiles (box), along with upper adjacent and lower adjacent values (whiskers)

Before matching, patients who received the US-ISB had a significantly lower i.v. morphine equivalent consumption at 2 postoperative hours (0.7 mg (CI 95% 0.1 to 1.2)) compared to control patients (8.8 mg (CI 95% 7.1 to 10.4); *P* < 0.0001; Fig. 1). After matching, the mean difference was 8.3 mg (95% CI 6.5 to 10.0), which remained significant (*P* < 0.001). The logistic regression analysis results indicated that none of the patients’ characteristics were associated with US-ISB group allocation, suggesting equivalent cohort selection for both the control and intervention groups (Additional file 1).

### Secondary outcomes

Tables 2 and 3 shows the secondary outcomes before and after the matching procedure respectively. All secondary outcomes were significantly lower in the US-ISB group, before and after the matching procedure, except resting pain scores and rate of PONV at 24 postoperative hours. Patients who received the US-ISB consumed significantly less Sufentanil intraoperatively with a mean difference of 28 μg (24-33 μg, *P* < 0.001). This also translated into lower pain scores for the US-ISB group in the PACU with a mean difference of 1.7 (0.8–2.5, *P* < 0.001) and lower morphine equivalent consumption at 24 h. Although the rate of PONV at 24 h did not retain a significant difference after the matching procedure, it is noteworthy to mention that 17% of patients who did not receive the US-ISB reported an episode of PONV at 24 h compared to 4% of patients who received the US-ISB. Balance checking results are provided in the Additional file 2.

**Table 2:** Secondary outcomes before matching. Data are presented as means and 95% confidence intervals

	Control group (*n* = 76)	US-ISB group (*n* = 50)	*p* value
Perioperative Sufentanil administration (μg)	45 (42–49)	17 (15–18)	< 0.001
Pain scores at rest at 2 postoperative hours (NRS, 0–10)	2.2 (1.8–2.6)	0.6 (0.2–1.1)	< 0.001
I.v. morphine equivalent consumption at 24 postoperative hours (mg)	16.7 (14.6–18.7)	6.9 (5.1–8.8)	< 0.001
Pain scores at rest at 24 postoperative hours (NRS, 0–10)	2.0 (1.5–2.4)	2.5 (1.9–3.1)	0.12
Rate of PONV within 24 postoperative hours	17%	4%	0.02

*NRS* numeric rating scale, *PONV* postoperative nausea and vomiting

**Table 3:** Secondary outcomes after matching. Data are presented as means with 95% confidence intervals

	Difference in means	*p* value
Perioperative Sufentanil administration (μg)	28 (24–33)	< 0.001 < 0.0001
Resting pain scores at 2 postoperative hours (NRS, 0–10)	1.7 (0.8–2.5)	< 0.001
I.v. morphine equivalent consumption at 24 postoperative hours (mg)	9.9 (6.7–13.0)	< 0.001
Resting pain scores at 24 postoperative hours (NRS, 0–10)	− 0.5 (− 0.4–1.3)	0.21
Rate of PONV within 24 postoperative hours	7% (− 3–17%)	0.23

*PONV* postoperative nausea and vomiting

## Discussion

This matched case-control cohort study investigated the analgesic efficacy of US-ISB for patients undergoing middle or lateral clavicle fracture ORIF. Our analyses showed that, when compared with patients who did not receive the regional procedure, patients with US-ISB received less intraoperative Sufentanil, consumed less opioid in i.v. morphine equivalents at 2 and 24 postoperative hours, and reported lower resting pain scores at 2 postoperative hours.

As summarized by Tran and colleagues, the clavicle may be innervated either by the supraclavicular nerve with its origin from the cervical plexus, or by the long thoracic nerve, the suprascapular nerve or even the subclavian nerve derived from the brachial plexus; a combined innervation from both plexuses is also possible [4]. We believe that our study brings clinically relevant evidence to this anatomic dilemma and, given the analgesic impact of US-ISB on postoperative analgesia after clavicle fracture ORIF, points towards a clavicle innervated at least in part by branches from the brachial plexus. The contribution of the cervical plexus remains unclear and further studies comparing analgesia provided with an ISB or a superficial cervical plexus block, or a detailed cadaveric study, may help to clarify remaining anatomic uncertainty.

### Limitations

Our study contains several limitations. First, this matched case-control cohort study suffers from the inherent weaknesses and potential biases of non-randomized interventions. Despite the inclusion of a detailed matching procedure, there may remain unknown confounding factors that might contribute to overestimation of the US-ISB’s analgesic efficacy during surgical fixation of middle or lateral clavicle fractures. We believe the likelihood of this is minimal given that our logistic regression analysis suggested equivalent allocation of patients across the two cohorts. Second, it could be argued that local anaesthetic may have spread from the interscalene groove towards the superficial cervical plexus, thereby limiting interpretation of our results. However, the US-ISB technique we adopted with an extrafascial needle tip location minimizes or eliminates the risk of spread towards the superficial cervical block, as recently demonstrated [8–10]. Finally, further exploration of the medial clavicle is needed given the middle/lateral distribution of fracture in this cohort.

## Conclusions

In conclusion, patients who received an US-ISB benefited from better analgesia after middle or lateral clavicle fracture ORIF, when compared with patients without US-ISB, and these results should help physicians establish an adequate analgesic strategy for managing this type of surgery. Further research is needed to identify the optimal regional technique for medial third clavicle fractures and the clinically relevant contributions of the cervical and brachial plexus.

## Supplementary information

**Additional file 1: Appendix 1**. Logistic regression analysis. Data are presented as log odds ratios with 95% confidence interval.

**Additional file 2: Appendix 2**. Balance checking: standardized difference in means.

## Data Availability

The datasets used and/or analysed during the current study are available from the corresponding author on reasonable request.

## Abbreviations

ASA: American society of Anesthesiologists score
ISB: Interscalene brachial plexus block
US-ISB: Ultrasound-guided interscalene brachial plexus block
ORIF: Open reduction and internal fixation
STROBE: Strengthening the Reporting of Observational Studies in Epidemiology
PACU: Post-Anesthesia Care Unit
NRS: Numeric rating scale
PONV: Postoperative nausea and vomiting
CI: Confidence intervals
