# Supplementary material for: Interscalene brachial plexus block for surgical repair of clavicle fracture: a matched case-controlled study
Source: BMC Anesthesiol. 2020 Apr 20;20:91. doi: 10.1186/s12871-020-01005-x (PMC7171737; doi:10.1186/s12871-020-01005-x)
Supplement: Supplementary file 1 — Additional file 1: Appendix 1. Logistic regression analysis. Data are presented as log odds ratios with 95% confidence interval. [file 12871_2020_1005_MOESM1_ESM.docx]

| Appendix 1. Logistic regression analysis. Data are presented as log odds ratios with 95% confidence interval. | |
| --- | --- |
| Gender | 0.09 (-0.94 – 1.12) |
| Age | 0.00 (-0.02 – 0.03) |
| Body mass index | 0.02 (-0.11 – 0.15) |
| ASA (2) | 0.04 (-0.72 – 0.81) |
| Fracture location (middle) | -0.32 (-1.16 – 0.50) |
| Constant | -0.29 (-1.58 – 0.98) |
