# Supplementary material for: Interscalene brachial plexus block for surgical repair of clavicle fracture: a matched case-controlled study
Source: BMC Anesthesiol. 2020 Apr 20;20:91. doi: 10.1186/s12871-020-01005-x (PMC7171737; doi:10.1186/s12871-020-01005-x)
Supplement: Supplementary file 2 — Additional file 2: Appendix 2. Balance checking: standardized difference in means. [file 12871_2020_1005_MOESM2_ESM.docx]

| Appendix 1. Balance checking : standardized difference in means. | |
| --- | --- |
| I.v. morphine equivalent consumption at 2 postoperative hours (mg) | |
| Gender | 0.09 |
| Age | -0.05 |
| Body mass index | -0.09 |
| ASA | 0 |
| Fracture location | -0.01 |
| Total dose of propofol at induction | 0.09 |
| Duration of surgery | 0.11 |
| Perioperative sufentanil administration (µg) | |
| Gender | 0.08 |
| Age | -0.05 |
| Body mass index | -0.09 |
| ASA | 0 |
| Fracture location | -0.01 |
| Total dose of propofol at induction | 0.09 |
| Duration of surgery | 0.12 |
| Resting pain scores at 2 postoperative hours (NRS, 0-10) | |
| Gender | 0.09 |
| Age | -0.09 |
| Body mass index | -0.11 |
| ASA | 0 |
| Fracture location | -0.01 |
| Total dose of propofol at induction | 0.13 |
| Duration of surgery | 0.15 |
| I.v. morphine equivalent consumption at 24 postoperative hours (mg) | |
| Gender | 0.08 |
| Age | -0.05 |
| Body mass index | -0.09 |
| ASA | 0 |
| Fracture location | -0.01 |
| Total dose of propofol at induction | 0.09 |
| Duration of surgery | 0.12 |
| Resting pain scores at 24 postoperative hours (NRS, 0-10) | |
| Gender | 0.07 |
| Age | -0.07 |
| Body mass index | -0.11 |
| ASA | 0 |
| Fracture location | -0.04 |
| Total dose of propofol at induction | 0.10 |
| Duration of surgery | 0.15 |
| Rate of PONV within 24 postoperative hours | |
| Gender | 0.08 |
| Age | -0.06 |
| Body mass index | -0.10 |
| ASA | 0 |
| Fracture location | -0.01 |
| Total dose of propofol at induction | 0.10 |
| Duration of surgery | 0.11 |
| PONV, postoperative nausea and vomiting | |
